# Apigenin Inhibits Human SW620 Cell Growth by Targeting Polyamine Catabolism

**DOI:** 10.1155/2017/3684581

**Published:** 2017-05-10

**Authors:** Jing Wang, TongMing Li, Linquan Zang, Xuediao Pan, Sujun Wang, Yanyan Wu, Guixiang Wang

**Affiliations:** ^1^School of Chinese Herbology, Guangzhou University of Chinese Medicine, Guangdong Province, Guangzhou, China; ^2^School of Pharmacy, Guangdong Pharmaceutical University, Guangdong Province, Guangzhou, China

## Abstract

Apigenin is a nonmutagenic flavonoid that has antitumor properties. Polyamines are ubiquitous cellular polycations, which play an important role in the proliferation and differentiation of cancer cells. Highly regulated pathways control the biosynthesis and degradation of polyamines. Ornithine decarboxylase (ODC) is the rate-limiting enzyme in the metabolism, and spermidine/spermine-N1-Acetyl transferase (SSAT) is the rate-limiting enzyme in the catabolism of polyamines. In the current study, the effect of increasing concentrations of apigenin on polyamine levels, ODC and SSAT protein expression, mRNA expression, cell proliferation and apoptosis, and the production of reactive oxygen species (ROS) was investigated in SW620 colon cancer cells. The results showed that apigenin significantly reduced cell proliferation, decreased the levels of spermidine and spermine, and increased previously downregulated putrescine contents. Apigenin also enhanced SSAT protein and mRNA levels and the production of reactive oxygen species in SW620 cells, though it had no significant effect on the levels of ODC protein or mRNA. Apigenin appears to decrease the proliferation rate of human SW620 cells by facilitating SSAT expression to induce polyamine catabolism and increasing ROS levels to induce cell apoptosis.

## 1. Introduction

Apigenin, a flavonoid commonly present in many edible fruits, vegetables, and Chinese herbs, is known to possess antitumor properties and thus has therapeutic potential for the treatment of cancer. A growing body of evidence suggests that apigenin exhibit antitumoral effects by retarding growth and inducing apoptosis through activation of pentose phosphate pathway-mediated NADPH generation in HepG2 human hepatoma cells [1]; induction of apoptosis via the PI3K/AKT and ERK1/2 MAPK pathways [2, 3]; decreasing the viability, adhesion, and migration of cancer cells [4, 5]; and modulating angiogenesis and metastasis [6]. Colorectal cancer (CRC) is the third most common cancer among men and the second most common among women worldwide. There are few treatment options for colon cancer; thus, preventative therapeutic approaches for this malignancy are necessary.

Data suggest that apigenin inhibits the proliferation of a wide range of cancer cells, including colorectal carcinoma cells, by inducing apoptosis [7]. Nevertheless, little is known about apigenin's interruption of certain cellular processes and whether this is involved in cancer inhibition.

**Figure 1:**
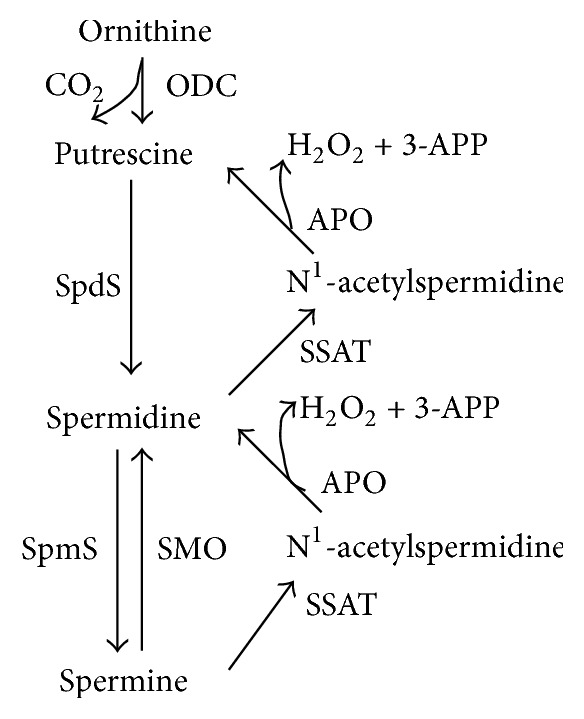
Polyamine metabolism. Ornithine decarboxylase (ODC) is required for the first step of polyamine synthesis in which ornithine is decarboxylated to produce putrescine. In turn, putrescine is converted sequentially to spermidine and spermine by the action of adenosylmethionine decarboxylase and spermidine and spermine synthases, respectively. The conversion to lower level polyamines is made in either two steps, by the spermidine/spermine N^1^-acetyltransferase (SSAT)/N^1^-acetylpolyamine oxidase (APAO) mechanism, or directly from spermine to spermidine by spermine oxidase (SMO). The activities of both APAO and SMO lead to the production of H_2_O_2_ and aldehydes 3-acetoaminopropanal (3-AAP) and 3-aminopropanal (3-AP).

Putrescine (Put), spermidine (Spd), and spermine (Spm) are polyamines. Polycationic polyamines are essential factors in mammalian development, and in eukaryotic cell differentiation and proliferation [8–12]. The concentration of cellular polyamines is typically low and is precisely controlled by metabolic modulation including polyamine uptake, transport, and interchange [8]; thus the intracellular concentration of polyamines is generally in the millimolar range. Polyamine metabolism (summarized in Figure 1) is strictly regulated under certain physiological conditions [13–15]. Ornithine decarboxylase (ODC), the rate-limiting enzyme of polyamine biosynthesis, plays an important role in polyamine generation, whereas spermidine/spermineN^1^-acetyltransferase (SSAT) is the rate-limiting enzyme of catabolism (Figure 1). Polyamines play vital roles in cell proliferation through association with nucleic acids, maintenance of chromatin conformation, regulation of specific gene expression, regulation of ion-channels, maintenance of membrane stability, and scavenging of free-radicals (especially spermidine) [16–21]. Previous research has demonstrated the mitogenic role of polyamines in enterochromaffin-like cells in health and disease [22]. Interestingly, high levels of polyamines are found in several types of tumors and play a unique role in cancer cell proliferation and survival [23]. The biological relationship between increased polyamine quantification and neoplasm growth has been well established. Specifically, hyperproliferative tumor cells have been shown to be accompanied by abnormally high polyamine levels [24–26]. Interest in the role of polyamines in hyperproliferative and cancerous tissues has led to the development of specific inhibitors for every step of polyamine metabolism, which has led to the development of several chemotherapeutic drugs. For example, treatment with the ornithine decarboxylase (ODC) inhibitor alpha-difluoromethylornithine (DFMO) results in the depletion of polyamines and inhibition of DNA synthesis, which consequently exerts a delay in cell cycle approaches [27–30]. Unfortunately, in most cancer cell lines, treatment with DFMO has a cytostatic rather than a cytotoxic effect [31–35]. In vitro studies consistently show that DFMO causes large increases in transmembrane polyamine uptake [36, 37].

**Figure 2:**
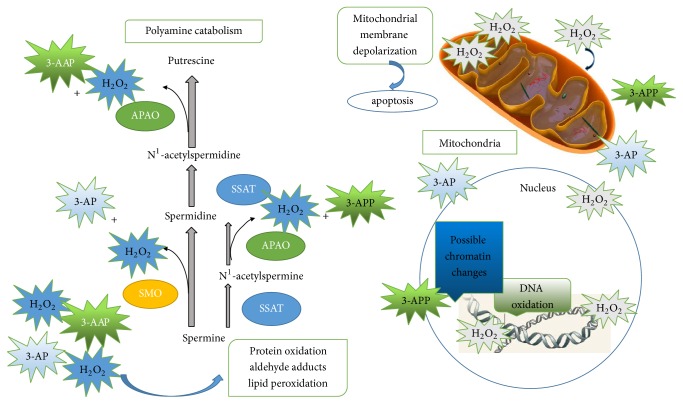
Potential contributions of polyamine catabolism to cell damage. Spermine is catabolized by spermine oxidase (SMO) to produce spermidine, 3-aminopropanal (3-AP), and H_2_O_2_. Spermine and spermidine can both be catabolized by spermidine/spermine N^1^-acetyltransferase (SSAT) to produce N^1^-acetylspermine and N^1^-acetylspermidine, which can then serve as substrates for N^1^-acetylpolyamine oxidase (APAO). This produces either spermidine or putrescine, respectively, along with 3-acetoaminopropanal (3-AAP) and H_2_O_2_. These reactive aldehydes and H_2_O_2_ are capable of damaging cellular machinery, lipids, and DNA, leading to cellular dysfunction or apoptosis.

Polyamine catabolic pathways of polyamines occur via a one-step process, directly from spermine to spermidine by spermine oxidase (SMO) or a two-step process via spermidine/spermine N^1^-acetyltransferase (SSAT)/N^1^-acetylpolyamine oxidase (APAO). Acetylated polyamines have two potential advantages: acetylated polyamine can be exported from the cell and acetylated spermidine and spermine are substrates for FAD-dependent, peroxisomal APAO. Spermidine or putrescine is the product of APAO, depending on the starting substrate, 3-acetoaminopropanal (3-AAP) or H_2_O_2_, respectively [38] (Figure 1). SSAT is an inducible enzyme, while APAO is generally constitutively expressed and rate-limited by the availability of the acetylated substrate. The coupled responses of SSAT and APAO, in particular, are responsible for preventing the overaccumulation of polyamines [39, 40]. It is worth noting that H_2_O_2_ is a by-product of the polyamine catabolic reaction, which is catalyzed by acetylpolyamine oxidase (APAO). It has been well established that H_2_O_2_, produced via APAO activity, is responsible for apoptosis [41]. Therefore, increases in SSAT expression produce increased acetylpolyamine derivatives, which are the preferred substrates for APAO. Furthermore, oxidation of these acetyl-polyamines by APAO produces H_2_O_2_, which then induces SSAT expression. Thus, the activities of APAO and SSAT activate the system that generates cellular death [16, 42]. This cycle of excessive acetylation and oxidation amplifies oxidative stress in the cell, thus producing a high local concentration of H_2_O_2_, possibly high enough to induce programmed cell death. ROS (H_2_O_2_ is typically one) at low levels refer to different signaling pathways. For example, activation of the NF-kB, ERK1/2, and PI3K pathways is regulated by low ROS levels and leads to an increase in cell growth and proliferation. By contrast, ROS at high levels may disrupt proper signaling pathways in the cell causing damage to DNA and other crucial macromolecules, which, in turn, may lead to DNA instability and consequently, senescence, or apoptosis [43, 44] (Figure 2).

The aim of this study was to determine the functional role of SSAT and polyamine catabolism in apigenin-treated colon cancer cells and to elucidate whether apigenin-induced polyamine catabolism, caused by an increase in SSAT activity, induced apoptosis via an increase in ROS production. The combination of polyamine biosynthesis and catabolism inhibition, via apigenin, may be a promising new therapeutic option for colon cancer and should be further studied in clinical trials in the future.

## 2. Materials and Methods

### 2.1. Chemicals, Reagents, and Antibodies

 CCK-8 kit (Yeasen), antibodies against anti-ODC, SSAT (NOVUS), *β*-actin (boster), secondary anti-mouse, anti-rabbit (Boster) DCFH-DA (Beyotime), Annexin V-FITC (KeyGEN BioTECH), Enhanced chemiluminescence (Thermo), Dulbecco modified Eagle medium (Gibco), foetal bovine serum (Gibco), Tyrisin (Gibco), PrimeScript^TM^ RT reagent kit with gDNA Eraser (Takara), SYBR® Premix Ex Taq^TM^ II (TaKaRa), Apigenin (Aladdin), DFMO (Sigma), and SPD (Sigma) were obtained.

### 2.2. Cell Culture Conditions

Human colon SW620 cells were obtained from the Institute of Biochemistry and Cell Biology, CAS (Shanghai, China). Cells were routinely cultured in Dulbecco modified Eagle medium (DMEM) supplemented with 10% foetal bovine serum (FBS) and equilibrated with humidified 5% CO_2_ in air at 37°C. Cells in the exponential phase of growth were used in experiments.

### 2.3. Proliferation Assay of SW620 Cells

2-(2-Methoxy-4-nitrophenyl)-3-(4-nitrophenyl)-5-(2,4-disulfophenyl)-2H-tetrazolium Sodium Salt (CCK-8) was used to measure SW620 cells proliferation. Cells were seeded onto 96-well tissue culture plates (5 × 10^3^ cells per well) in Dulbecco modified Eagle medium with 10% FBS. Cells were cultured in serum-free Dulbecco modified Eagle medium for 24 hours, and different concentrations of apigenin (10 *μ*M, 20 *μ*M, 40 *μ*M, and 80 *μ*M) and DFMO (2.5 mM) were then added. After 24, 48, and 72 hours of incubation, the cells were treated with 10 *μ*l WST-8 per well for 4 hours at 37°C in humidified CO_2_. WST-8 conversion to formazan by metabolically viable cells was monitored using a spectrophotometer at an optical density of 450 nm.

### 2.4. Polyamine Analysis

To determine cellular polyamine content, 1 × 10^6^ cells were seeded on 60 mm Petri dishes and allowed to attach overnight. Cells were then treated with DFMO (2.5 mM), SPD (0.5 *μ*M), apigenin (10 *μ*M, 20 *μ*M, and 40 *μ*M), or apigenin (10 *μ*M, 20 *μ*M, and 40 *μ*M) in combination with DFMO (2.5 mM).

Cells were harvested after 48 h treatment and 1 ml of trichloroacetic acid (5%) was added to each sample to precipitate the protein. Samples were immediately run on HPLC (Shimadzu) after benzoylation processing to determine benzoyl-polyamine derivatives. Polyamine levels are expressed as concentration values in nmol/mg of protein.

### 2.5. Western Blot Analysis

Expression levels of proteins involved in polyamine metabolism (ODC, SSAT) were analyzed by Western blot and normalized to the mean expression of *β*-actin as described in a previous study.

SW620 cells were cultured with 10 *μ*M, 20 *μ*M, or 40 *μ*M of apigenin and DFMO (2.5 mM). After 48 h treatment, the cells were washed twice in ice-cold 1x PBS and resuspended in a buffer consisting of 20 mM Tris-HCl (pH 7.4), 150 mM NaCl, 1 mM EDTA (pH 8.0), 1% Triton X-100, and 0.1% SDS, supplemented with complete protease inhibitor cocktail (Beyotime). The lysates were then centrifuged at 12000 rpm for 25 minutes at 4°C. The supernatant was used for protein estimation using a BCA protein assay kit (Thermo). Approximately, 40–60 *μ*g of protein was loaded into each well of SDS-gel (10% or 12%) and transferred onto a PVDF (minipall) membrane. The blots were then blocked in 5% milk in TBST (Tris buffered saline-tween 20) for 1 h and incubated overnight at 4°C with the relevant primary antibodies (1 : 1000) diluted in 5% milk in TBST. Following this, HRP-conjugated secondary antibodies (1 : 5000), diluted in 5% milk/TBST, were added and incubated for 1 hour, washed with 1x TBST and developed using ECL detection reagent (Thermo). For sequential antibody reprobing, blots were stripped using 1x TBST. Band densities were measured using image J software and results were normalized to corresponding loading controls.

### 2.6. Real-Time Reverse Transcription-PCR Analysis

The total RNA of the cells was isolated using RNAiso Plus, following the manufacturer's protocol. First-strand cDNA was synthesized with a PrimeScript II 1st Strand cDNA Synthesis Kit, using 3 *μ*l of total RNA and 10 pmol of primers, according to the manufacturer's instructions (Table 1). Real-time PCR was performed with SYBR Premix Ex Taq II, following the manufacturer's protocol. Gene expression levels were normalized to those of GAPDH.

### 2.7. Determination of Generation of Reactive Oxygen Species

The intracellular formation of reactive oxygen species (ROS) was measured using 7′-dichlorodihydrofluorescin diacetate (DCFH-DA) (Beyotime). The nonfluorescent compound, DCFH-DA, penetrates into the cell and is cleaved by intracellular esterases, resulting in the formation of 2′,7′-dichlorodihydrofluorescin (DCFH), the oxidation of which (due to oxidative stress) generates the fluorescent compound dichlorofluorescein. Thus, DCF fluorescence represents the rate and quantity of ROS produced. After the Api treatment procedure, cells were stained with 1 *μ*M DCFH-DA at 37°C for 30 min and washed twice with PBS and the fluorescence was measured using an FCM (BD Bioscience).

### 2.8. Apoptotic Assay

The Annexin V-FITC Apoptosis Detection (KeyGEN) was used to assess cell apoptosis induced by apigenin treatment. SW620 cells were cultured in 60 mm Petri dishes and allowed to grow overnight. The cells were then treated with AP (10 *μ*M, 20 *μ*M, and 40 *μ*M), DFMO (2.5 mM), or DFMO (2.5 mM) in combination with AP (40 *μ*M). After 48 h of treatment, cells were harvested and washed twice with PBS, resuspended with 500 *μ*l binding buffer, and stained with 5 *μ*l Annexin V-FITC and 5 *μ*l PI for 15 min at the room temperature in the dark. Cells were analyzed by FCM (BD).

### 2.9. Statistical Analysis

All data was statistically analyzed by two-tailed unpaired Student's *t*-test and two-way ANOVA using GraphPad Prism version 5.04 for Windows, Graph Pad Software (La Jolla, California, USA). Error bars in the graphs were generated using ± standard deviation (SD) values. A probability of *p* < 0.05 was considered significant for all statistical analysis.

## 3. Results

### 3.1. Effect of Different Concentrations of Apigenin on the Growth of SW620 Cells

**Figure 3:**
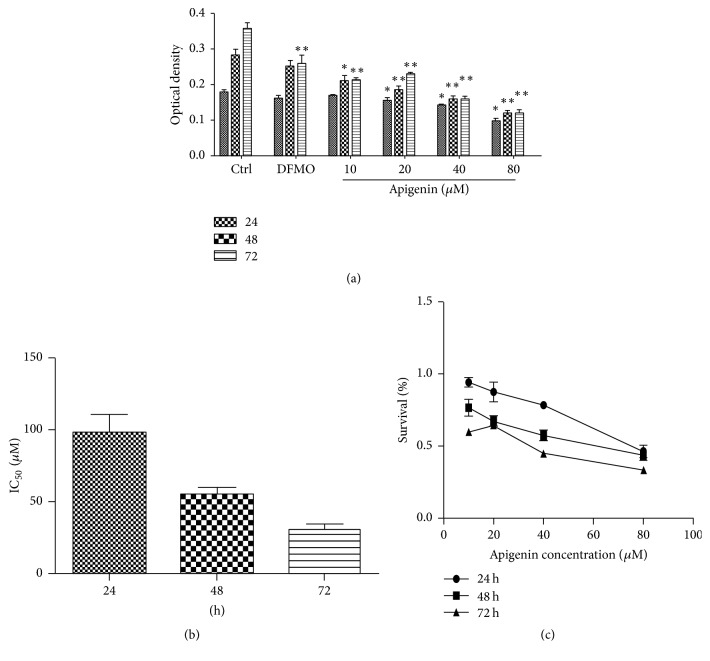
(a) Effect of increasing concentrations of apigenin and DFMO (2.5 mM) on the conversion of CCK8 Sodium Salt in SW620 cells. (c) The dose-response curve of apigenin and (b) the comparison of the IC_50_ values (*μ*M) for apigenin in SW620 cells were determined after 24-, 48-, and 72-hour exposure to the drugs. The IC_50_ is defined as the concentration causing 50% growth inhibition in treated cells compared to controls. All data represent the results of 6 different experiments (mean value ± SD). The *p* value was determined by one-way analysis with Dunnett's posttest. ^*∗*^*p* < 0.05 and ^*∗∗*^*p* < 0.01 versus control.

To determine the effect of increasing concentrations of apigenin and DFMO on SW620 cell proliferation, cell viability was measured using 2-(2-Methoxy-4-nitrophenyl)-3-(4-nitrophenyl)-5-(2,4-disulfophenyl)-2H-tetrazolium Sodium Salt (CCK-8). SW620 cells were treated for 24, 48, or 72 hours with apigenin concentrations ranging from 10 *μ*M to 80 *μ*M, and DFMO (2.5 mM) was used as a positive control (Figure 3(a)). After 48 and 72 hours, apigenin significantly reduced the cell proliferation compared to untreated control cells, while DFMO caused a slight reduction in cell viability compared to apigenin, indicating that apigenin is more selective than traditional chemotherapeutic compounds (Figure 3(c)). The IC_50_ of apigenin was 88.3 ± 12.3 *μ*M at 24 hours, 54.9 ± 8.1 *μ*M at 48 hours, and 30.6 ± 6.1 *μ*M at 72 hours (Figure 3(b)). As is evident, 72 hours was the most effective of the three periods of time.

### 3.2. Modulation of ODC and SSAT Protein Expression by Apigenin and DFMO

**Figure 4:**
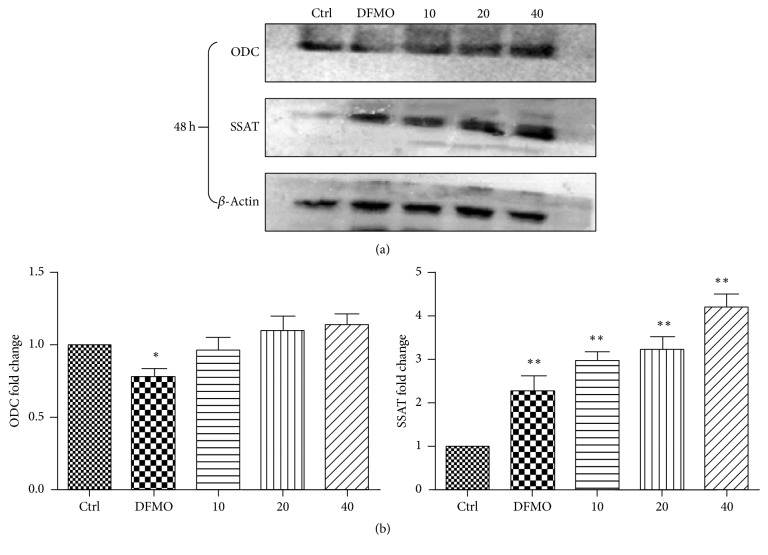
Western blot analysis and quantification. (a) Western blot analysis of ODC and SSAT protein levels in cells treated with apigenin (10, 20, and 40 *μ*M) and DFMO (2.5 mM) for 48 hours. Twenty-five micrograms of total protein was loaded per lane. SSAT activity was observed at 48 h. There was no significant activation of ODC after apigenin treatment at either concentration. (b) Quantification of ODC and SSAT expression in SW620 cells treated with apigenin (10, 20, and 40 *μ*M) and DFMO (2.5 mM) at 48 h. ^*∗*^*p* < 0.05 and ^*∗∗*^*p* < 0.01 relative to untreated cells.

Western blot analysis (Figure 4) was used to show that ODC protein levels were not affected by apigenin (10, 20, and 40 *μ*M), while DFMO (2.5 mM) significantly reduced the ODC protein levels in SW620 cells. These results indicate that ODC may not be the target of apigenin. It is interesting to note that SSAT protein levels were upregulated with apigenin treatment (Figures 3(a) and 3(b)).

### 3.3. Apigenin Treatment Affects SSAT mRNA Expression in SW620 Cells

**Figure 5:**
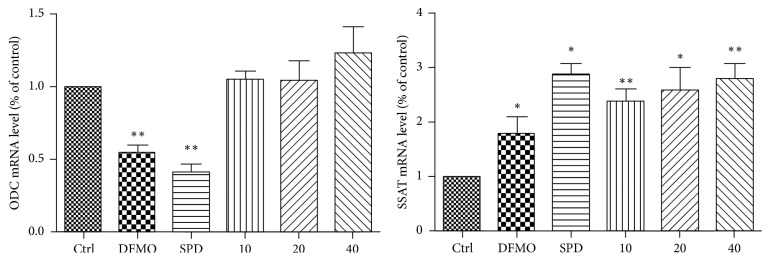
RT-qPCR analysis of ODC and SSAT mRNA expression in SW620 after 48 h of treatment with apigenin (10, 20, and 40 *μ*M), DFMO (2.5 mM), and exogenous-spermidine (5 *μ*M). RT-qPCR data are presented as fold change compared to untreated. The *p* value was determined by one-way analysis with Dunnett's posttest. ^*∗*^*p* < 0.05 and ^*∗∗*^*p* < 0.01 relative to untreated cells.

In order to validate the mechanism by which apigenin affects ODC and SSAT, ODC and SSAT mRNA levels were studied after treatment with apigenin (10, 20, and 40 *μ*M), DFMO (2.5 mM), and exogenous-spermidine (5 *μ*M) for 48 h. Real-Time PCR analysis (Figure 5) demonstrated a significant increase in SSAT mRNA expression in response to apigenin treatment, while there were no significant changes in ODC mRNA levels after treatment with apigenin. Interestingly, exogenous-spermidine increased SSAT mRNA levels and decreased ODC mRNA levels, indicating that polyamine metabolism is homeostatic. This homeostasis was possibly disrupted by apigenin through promotion of polyamine catabolism by targeting SSAT.

### 3.4. Treatment with Apigenin Resulted in Acceleration of Cellular Polyamine Catabolism and Treatment with DFMO and Apigenin Combined Resulted in Depletion of Cellular Polyamine Levels

Further research was done to investigate cellular polyamine content after 48 hours of treatment with apigenin (10, 20, and 40 *μ*M), DFMO (2.5 mM), and combination of DFMO and apigenin (10, 20, and 40 *μ*M).

**Figure 6:**
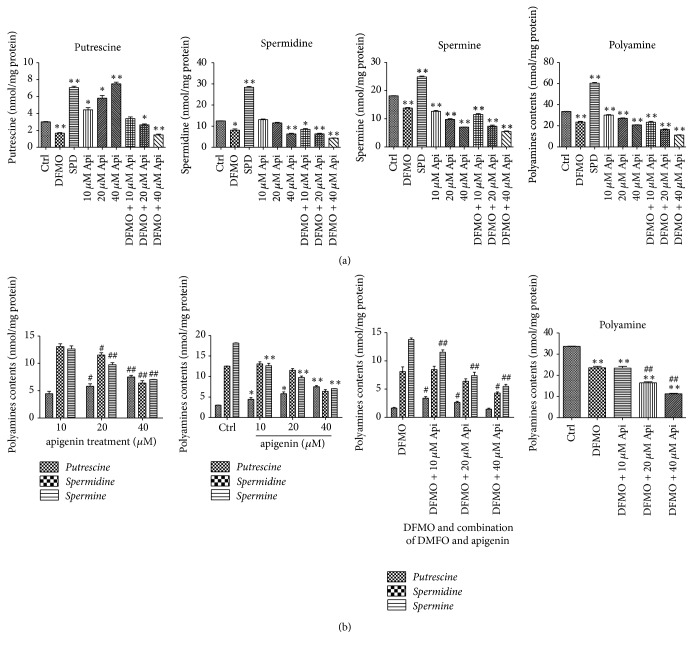
(a) Analysis of putrescine, spermidine, spermine, and total polyamine contents (nmol/mg protein) in SW620 cells treated with apigenin (10, 20, and 40 *μ*M), exogenous-spermidine (5 *μ*M), DFMO (2.5 mM), and a combination of DFMO and apigenin (10, 20, and 40 *μ*M) for 48 h. Total polyamine contents were decreased by both apigenin and DFMO. Furthermore, the combination of apigenin and DFMO induced an even greater decrease in polyamine levels. ^*∗*^*p* < 0.05 and ^*∗∗*^*p* < 0.01 versus control. (b) Cells exposed to increased concentrations of apigenin demonstrate a concentration-dependent increase in putrescine level and decrease in spermine level with a decrease in total polyamine content. Accordingly, intergroup and intragroup polyamine levels were analyzed. ^#^*p* < 0.05 and ^##^*p* < 0.01 compare doses; ^*∗*^*p* < 0.05 and ^*∗∗*^*p* < 0.01 versus control. (c) In order to investigate the effect of a combination of DFMO and apigenin on polyamine levels, increasing concentrations of apigenin were combined with DFMO. Total polyamine content was analyzed by intergroup comparison. ^#^*p* < 0.05 and ^##^*p* < 0.01 compare doses; ^*∗*^*p* < 0.05 and ^*∗∗*^*p* < 0.01 versus control.

Treatment with apigenin, DFMO, and a combination of apigenin DFMO a decreased specific intracellular polyamines, while polyamine contents were greatly increased by treatment with exogenous-spermidine (SPD) (Table 2 and Figure 6(a)). It is interesting to note that DFMO treatment alone almost completely depleted putrescine levels and significantly decreased the levels of spermidine and spermine in SW620 cells, while apigenin treatment resulted in a concentration-dependent increase in putrescine levels and decrease in spermine levels with a decrease in the level of total polyamine content (Figures 6(a) and 6(b)), indicating that polyamine catabolism is promoted by apigenin to some extent. Furthermore, combined treatment with DFMO and increasing concentrations of apigenin resulted in apigenin concentration-dependent depletion of cellular polyamine levels (Figure 6(b)). These results indicate there was a synergistic effect with the combined treatment of DFMO and apigenin, thus suggesting that apigenin contributes to polyamine catabolism by targeting SSAT. The reason for this is that SSAT is an inducible enzyme, while APAO (acetylpolyamine oxidase) is generally constitutively expressed and rate-limited by the availability of the acetylated substrate (Figure 1).

### 3.5. Apigenin Treatment, as well as Treatment with a Combination of DMFO and Apigenin, Increased Cellular ROS Levels

**Figure 7:**
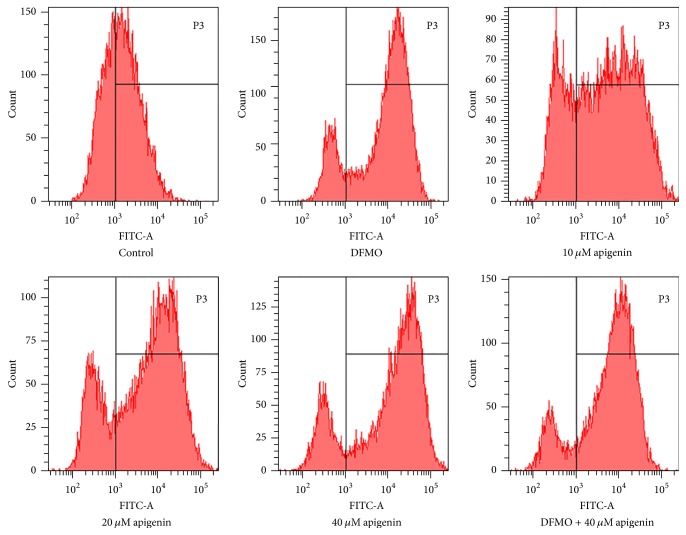
Cellular ROS level analysis. ROS levels were significantly increased in SW620 cells treated with apigenin (10, 20, and 40 *μ*M), DFMO (2.5 mM), or combination of 5 mM DFMO and 40 *μ*M apigenin.

To investigate whether apigenin augmented ROS induced by overexpression of SSAT in SW620 cells, cells were treated with 2.5 mM DFMO, apigenin at 10, 20, and 40 *μ*M, or a combination of 2.5 mM DFMO and 40 *μ*M apigenin for up to 48 h. ROS level analysis revealed that exposure to 2.5 mM DFMO resulted in a significant increase in cellular ROS (*p* < 0.01). There was a 1- to 1.3-fold increase in the percentage of ROS levels, with the addition of increasing apigenin concentrations, versus control. DFMO combined with 40 *μ*M apigenin produced a 1- to 1.5-fold increase in ROS levels compared to control (*p* < 0.01) (Figures 7 and 9).

### 3.6. Apigenin Treatment Resulted in Apoptotic Cell Death of SW620 Cells and Treatment with DFMO and Apigenin Further Increased Apoptosis

**Figure 8:**
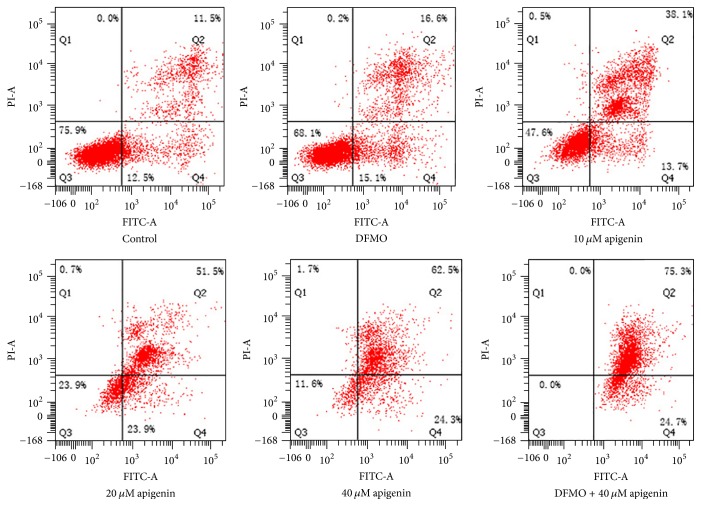
Apoptosis analysis and quantification. Apoptosis was significantly induced in SW620 cells after treatment with apigenin (10, 20, and 40 *μ*M), DFMO (5 mM), or combination of 5 mM DFMO and 40 *μ*M apigenin.

**Figure 9:**
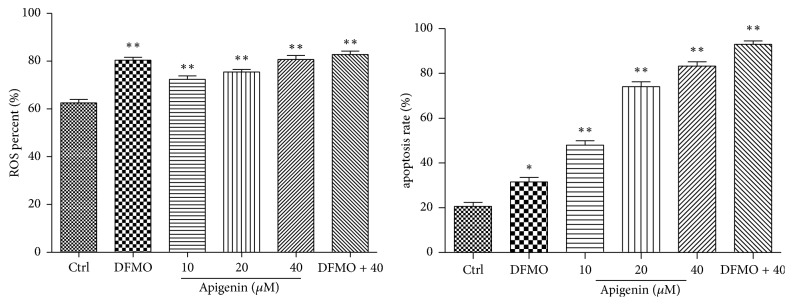
Quantification of ROS levels and cell apoptosis. ^*∗*^*p* < 0.05 and ^*∗∗*^*p* < 0.01 versus control.

To further validate the effects of apigenin and DFMO on cell apoptosis, SW620 cells were treated with 2.5 mM DFMO, 10, 20, and 40 *μ*M apigenin, or combination of 2.5 mM DFMO and 40 *μ*M apigenin for 48 h. Treatment led to induction of apoptosis as measured by the flow cytometry. The treatment promoted a concentration-dependent apoptosis, whereas a combination of DFMO and 40 *μ*M apigenin had a more evident effect on apoptosis compared to untreated cells (Figures 8 and 9).

## 4. Discussion

The natural flavonoid apigenin has shown antiproliferative activity against a variety of human cancer cells [1–4] and it has also been suggested as an anticancer drug for the treatment of colon cancer since it has been shown to inhibit the proliferation of human colon tumor HCT-116 cells [5].

Polyamine catabolism produces hydrogen peroxide and a reactive aldehyde, which are able to damage DNA and other critical cellular components. The catabolic pathway also depletes intracellular concentrations of spermidine and spermine, which are free radical scavengers (Figure 2). SSAT is a key rate-limiting enzyme in mammalian polyamine catabolism, which can be induced by various stimuli, like natural polyamines, polyamine analogues, hormones and cytokines, drugs, and so on [45–47]. Therefore, an increase in the activity of SSAT may be an initiating signal for the cessation of cell growth and proliferation. Indeed, this is the key enzyme for regulating the response of polyamine for downregulation of growth for the short half-life of this enzyme and its inducibility in response to stimuli. Other polyamine acetyltransferases are present in eukaryotic cells, including acetyltransferase, with specificity for the N8-amino group of spermidine, but the activity of this enzyme dose did not change significantly in a previous in vitro study and seems to be of less importance for the regulation of cell growth [48].

To the best of our knowledge, this is the first study to explore the effect of apigenin on polyamine catabolism by targeting spermidine/spermine-N1-Acetyltransferase and induction of apoptosis. Data from the present study demonstrates that apigenin treatment for 24 h–72 h influenced the proliferation rate of SW620 cells in a dose -dependent manner (Figure 3). SSAT protein and mRNA levels significantly increased after exposure to apigenin for 48 h (Figures 4 and 5). The acceleration of polyamine catabolism by apigenin treatment was further supported by HPLC data (Figure 6). Treatment with different concentration of apigenin resulted in an acceleration of cellular polyamine catabolism in a dose dependent manner, which was manifested in the increase of putrescine and decrease of spermine and spermidine levels. Combined treatment with DFMO and apigenin resulted in a depletion of cellular polyamine levels. When investigating the mechanisms behind the effect of apigenin treatment on polyamine levels, a great increase in ROS levels was observed in SW620 cells (Figure 7). This suggests strongly that SSAT is responsible for the induction of ROS and that an increase in SSAT is an early indication of potential cellular injury. Subsequent apoptotic analysis further indicated that the apigenin-induced SSAT activity was associated with cell death by apoptosis (Figure 8). Taken together, these results indicate that apigenin may affect the growth of human SW620 cells and decrease the rate of cell proliferation by facilitating SSAT expression to induce polyamine catabolism and increase ROS levels to induce cell apoptosis.

In summary, apigenin treatment induces a rise in the expression of SSAT in human SW620 colon cancer cells. This increase may be an initiating signal for apoptosis and is likely to be destructive to the cell in that it will catabolize polyamine to act as a cellular ROS scavenger. Increased SSAT expression will increase the acetylpolyamine derivatives produced; since these were not detected within the cells they are presumably further metabolized or released into the extracellular medium [49]. Considering the ability of SSAT to reduce the cellular concentrations of the free radical scavenging higher polyamines, as well as producing a reactive aldehyde and H_2_O_2_ that can result in mutagenic DNA damage or chromatin changes, polyamine catabolism could be a promising target for chemoprevention or chemotherapy. The effect of apigenin on polyamine catabolism warrants further investigation.

## Figures and Tables

**Table 1:** Amplification primer sequences used.

Gene	Primer
ODC	Forward: 5′-GGGCTGGGTTGGTTTCA-3′
	Reverse: 5′-ACGCTGGGTTGATTACGC-3′
SSAT	Forward: 5′-CAGTGACATACTGCGGCTGA-3′
	Reverse: 5′-GTGCTCTTTCGGCACTTCTG-3′
GAPDH	Forward: 5′-AACGGATTTGGTCGTATTGGG-3′
	Reverse: 5′-CCTGGAAGATGGTGATGGGAT-3′

**Table 2:** Polyamine content (nmol/mg protein) of SW620 cells after exposure to different concentrations of apigenin (10, 20, and 40 *μ*M), DFMO (2.5 mM), and a combination of DFMO and apigenin for 48 h. The effect of exogenous-spermidine (5 *μ*M) after 48 h of treatment is also shown. The *p* value was determined by one-way analysis with Dunnett's posttest. ^*∗*^*p* < 0.05 and ^*∗∗*^*p* < 0.01 versus control.

Treatment	Growth parameter (nmol/mg protein)			
	Putrescine	Spermidine	Spermine	Total polyamines
Ctrl	3.01 ± 0.05	12.53 ± 0.02	18.15 ± 0.07	33.69 ± 0.02
DFMO	1.66 ± 0.12^*∗∗*^	8.12 ± 0.82^*∗*^	13.78 ± 0.29^*∗∗*^	23.55 ± 0.97^*∗∗*^
SPD	7.06 ± 0.23^*∗∗*^	28.37 ± 0.65^*∗∗*^	24.88 ± 0.41^*∗∗*^	60.37 ± 1.17^*∗∗*^
10 *μ*M Api	4.44 ± 0.46^*∗*^	13.07 ± 0.52	12.64 ± 0.56^*∗∗*^	30.15 ± 0.96^*∗∗*^
20 *μ*M Api	5.81 ± 0.44^*∗*^	11.53 ± 0.42	9.77 ± 0.36^*∗∗*^	27.12 ± 0.58^*∗∗*^
40 *μ*M Api	7.52 ± 0.27^*∗∗*^	6.39 ± 0.43^*∗∗*^	7.04 ± 0.03^*∗∗*^	20.86 ± 0.26^*∗∗*^
DFMO + 10 *μ*M Api	3.40 ± 0.28	8.47 ± 0.60^*∗*^	11.53 ± 0.42^*∗∗*^	23.40 ± 1.14^*∗∗*^
DFMO + 20 *μ*M Api	2.66 ± 0.18^*∗*^	6.39 ± 0.44^*∗∗*^	7.39 ± 0.61^*∗∗*^	16.44 ± 0.88^*∗∗*^
DFMO + 40 *μ*M Api	1.46 ± 0.17^*∗∗*^	4.31 ± 0.21^*∗∗*^	5.50 ± 0.33^*∗∗*^	11.27 ± 0.50^*∗∗*^
